# The burdens of lung cancer involved multiple primary cancers and its occurring patterns–SEER Analysis between 1973 and 2006

**DOI:** 10.1038/s41598-017-06763-2

**Published:** 2017-07-25

**Authors:** Rui Mao, Tao Chen, Fangyu Zhou, Weili Jiang, Xiaorong Yang, Zisheng Ai, Mu Li, Linlin Qin, Long Wang, Ke Fei, Chang Chen

**Affiliations:** 1 Department of Thoracic Surgery, Shanghai Pulmonary Hospital, Tongji University School of Medicine, Shanghai, PR China; 2 Department of Epidemiology, School of Public Health, Fudan University, Shanghai, PR China; 3 Key Laboratory of Public Health Safety (Ministry of Education), Shanghai, PR China; 4 Department of Epidemiology and Biostatistics, Shandong University, Shandong, PR China; 5 Department of Medical Statistics, TongJi University School of Medicine, Shanghai, PR China

## Abstract

The prognosis of malignancies has improved in recent years, subsequent primary cancers (SPCs) have become more frequent. This study investigates the patterns of lung cancer involved multiple primary cancers. We enrolled 206,619 primary lung cancer patients and 2,071,922 patients with other primary malignancies from Surveillance, Epidemiology and End Results (SEER) database. Observed annual risk (OAR) and absolute numbers were used to describe the risk of SPC and observed cases of SPC per 10,000 person-years at risk. Overall, OAR of SPCs following lung cancer was 176.28. At follow-up, 41.26% of SPCs occurred within 12–59 months while the highest OAR appeared after 120 months. The overall OAR of subsequent lung cancer after other malignancies was 27.90. Overall, the highest OAR and the highest absolute numbers of subsequent lung cancers were noticed 60–119 months and over 120 months post-diagnosis, respectively. Ten related cancers were listed. Our findings encourage surveillance for 10 common SPCs in lung cancer survivors during follow-up as well as screening for lung cancer after 10 common malignancies.

## Introduction

With the rapid advancements in diagnosis and treatment, the survival times of cancers have become longer in recent years, and subsequent primary cancers (SPCs) have become increasingly common in the investigations of clinical scientists^1–3^. In 2015, lung cancer was the second most common cancer and also the leading cause of mortality in the United States^4^. For patients who received curative surgery, the 5-year survival rate increased to 47–58%^5–7^. In the meantime, 1.5–4.7% of lung cancer survivors developed SPCs during their follow-up time^8–10^. Similarly, following other primary malignancies, subsequent lung cancers were also reported^11^. However, previous studies have tended to discuss the incidence ratio of SPCs, rather than illustrating the patterns of actual burden for these multiple primary cancers. To depict the patterns of burden, we assessed the influences of first primary cancers on the incidence of SPCs based on the Surveillance, Epidemiology and End Results (SEER) database between 1973 and 2006.

## Method

### Study population

Since 1973, the National Cancer Institute’s SEER program has collected high-quality, population-based data on cancer incidence and survival in the United States. All cancers occurring among residents of defined geographical registries comprising the SEER program are reportable. The current cohort comprised patients diagnosed with cancer between January 1973 and December 2006 in 9 original registries (Atlanta, Connecticut, Detroit, Hawaii, Iowa, New Mexico, San Francisco-Oakland, Seattle-Puget Sound, and Utah) retrieved from the “Incidence-SEER 9 Regs Research Data, Nov 2015 Sub (1973–2013) <Katrina/Rita Population Adjustment>” database, which covers approximately 9.4% of the US population (based on 2010 census). Excluded cases were those of patients 1) without microscopic confirmation or for whom the report had been obtained solely from a death certificate or autopsy report, 2) whose follow-up time was less than 6 months, and 3) having multiple lung and bronchus cancers.

### Ethics statement

This study was conducted in compliance with the Helsinki Declaration and approved by an independent ethics committee/institutional review board at Shanghai Pulmonary Hospital. The methods were carried out in accordance with the approved guidelines in this study. Data released from the SEER database do not require informed patient consent because they contain no identifiers and are publicly available. We obtained permission to access the research data file in the SEER program by National Cancer Institute, USA, under the reference number 10274-Nov 2015.

### Definitions of AER, OAR and EAR

Similar to annual excess risk (AER), which is calculated as the cases (observed–expected) in excess of SPCs per 10,000 person-years at risk (PYR), observed annual risk (OAR) indicates the absolute number of SPCs in patients with a specific primary malignancy per 10,000 PYR^12^. Expected annual risk (EAR) was also used to represent the expected cases of SPCs per 10,000 PYR. Expected number of specific primary cancers (first and subsequent) was evaluated for a reference SEER cohort after adjusting for age, gender, ethnicity, and calendar year period.

### Statistical Analysis

The latency period of SPCs begins 6 months after the initial cancer diagnosis and ends with SPC diagnosis. The OARs, EARs, and AERs of SPCs after lung and bronchus cancer were calculated to explore the patterns of primary lung and bronchus cancer that contributed to the development of other subsequent malignancies. Similar calculations were performed to evaluate the incidence of subsequent lung and bronchus cancers following other primary cancers. OAR, EAR, AER, and PYR values were calculated using Multiple Primary-Standardized Incidence Ratios (MP-SIR) in SEER*Stat software (Release 8.3.2, 2016; National Cancer Institute Cancer Statistics Branch, Bethesda, MD).

## Results

### Demographics

Overall, 206,619 primary lung and bronchus cancer patients were enrolled. Within a median follow-up time of 99 months, 10,694 patients (5.18%) were diagnosed with 11,891 SPCs. Median age at first diagnosis was 65 years old. In the cohort of lung and bronchus cancer cases with SPCs, most patients were male (64.12%) (n = 6,857) and White (84.77%) (n = 9,065). Table 1 summarizes the characteristics of the patients with SPCs.

**Table 1:** Characteristics of patients with SPCs.

Characteristics	First cancer site			
	LBC		All sites excluding LBC	
	No. (N = 10,694)	% (100%)	No. (N = 54,897)	% (100%)
Sex				
Male	6,857	64.12	33,431	60.90
Female	3,837	35.88	21,466	39.10
Race				
White	9,065	84.77	47,564	86.64
Black	1,096	10.25	4,957	9.03
Other	533	4.98	2,376	4.33
Follow-up time, month				
6–11	358	3.35	653	1.19
12–59	3,022	28.26	13,113	23.89
60–119	2,879	26.92	16,584	30.21
120+	4,435	41.47	24,547	44.71
Median	99		108	
Range	6–48		6–490	
Q1	45		59	
Q3	168		174	
Age at first diagnosis, year				
Median	65		65	
Range	16–93		1–99	
Q1	58		57	
Q3	71		71	

Abbreviations: SPC, subsequent primary cancer; LBC, lung and bronchus cancer; Q, quartile.

Reciprocally, among 2,071,922 survivors of other malignant tumors, a total of 56,479 lung and bronchus cancers occurred in 54,897 (2.65%) individuals during a median follow-up time of 108 months. Similar to the former cohort, 60.90% (n = 33,431) of these patients were male, and 86.64% (n = 47,564) were White. The median age at first diagnosis was 65 years old (Table 1).

### SPCs following lung and bronchus cancer

As shown in Table 2, the overall OAR of SPCs was 176.28 per 10,000 PYR. The OAR was 212.27 per 10,000 PYR in males (n = 7,659, OAR = 212.27, AER = 30.05), and 134.88 per 10,000 PYR in females (n = 4,232, AER = 16.97).

**Table 2:** OARs, EARs, and AERs of SPCs after LBCs.

	SPCs after LBC in all patients			SPCs after LBC in male patients			SPCs after LBC in female patients		
	Absolute numbers	OARs	AERs	Absolute numbers	OARs	AERs	Absolute numbers	OARs	AERs
		EARs			EARs			EARs	
All cancers excluding LBC	11,891	176.28	23.97	7,659	212.27	30.05	4,232	134.88	16.97
		152.31			182.22			117.91	
Prostate	2,213	61.33	−7.87	2,213	61.33	−7.87	—	—	—
		69.20			69.20				
Colorectum	1,832	27.16	4.81	1,122	31.10	5.3	710	22.63	4.25
		22.35			25.80			18.38	
Breast	1,223	18.13	−0.01	21	0.58	0.14	1,202	38.31	−0.18
		18.14			0.44			38.49	
Urinary Bladder	1,162	17.23	6.83	909	25.19	9.27	253	8.06	4.01
		10.40			15.92			4.05	
Oral Cavity and Pharynx	646	9.58	5.30	473	13.11	7.13	173	5.51	3.19
		4.28			5.98			2.32	
Non-Hodgkin Lymphoma	476	7.06	0.32	289	8.01	0.42	187	5.96	0.20
		6.74			7.59			5.76	
Pancreas	456	6.76	1.70	268	7.43	1.86	188	5.99	1.52
		5.06			5.57			4.47	
Kidney and Renal Pelvis	431	6.39	1.82	286	7.93	2.00	145	4.62	1.62
		4.57			5.93			3.00	
Leukemia	411	6.09	1.14	247	6.85	0.67	164	5.23	1.70
		4.95			6.18			3.53	
Larynx	392	5.81	3.96	305	8.45	5.43	87	2.77	2.25
		1.85			3.02			0.52	

Abbreviations: SPC, subsequent primary cancer; LBC, lung and bronchus cancer; OAR, observed annual risk; EAR, expected annual risk; AER, Annual excess risk.

**Figure 1:**
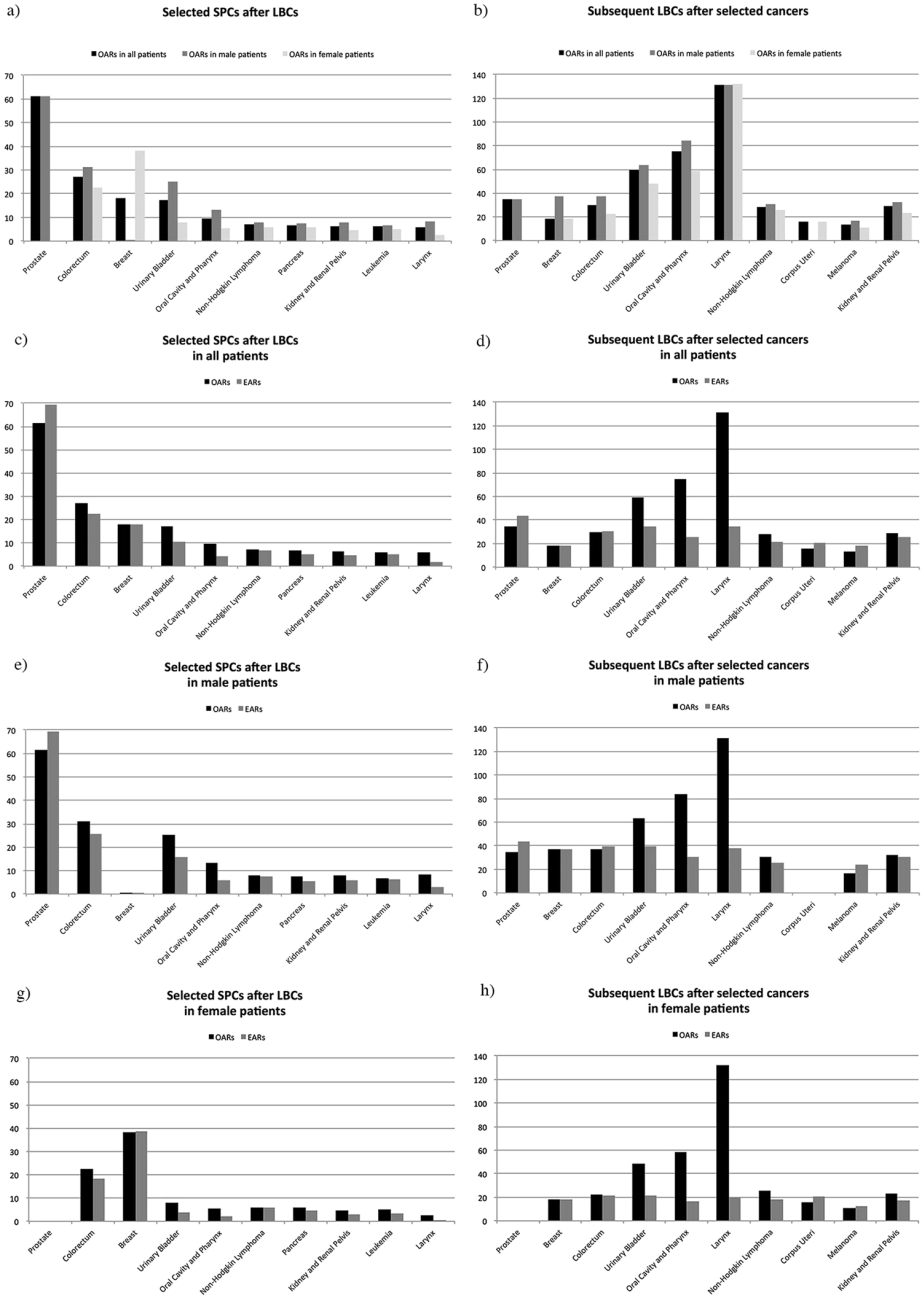
Observed annual risks (OARs) and expected annual risks (EARs) of selected subsequent primary cancers (SPCs) after LBCs and subsequent LBCs after selected cancers, by gender.

Table 2 and Fig. 1 describe the OARs and EARs for the 10 most common SPCs after lung and bronchus cancer. The observed incidence, as measured by OAR, ranged from low OAR for larynx (n = 392, OAR = 5.81, AER = 3.96) to high OAR for prostate cancer (n = 2,213, OAR = 61.33, AER = −7.87). Stratification by gender revealed that OARs of all selected SPCs were higher in males than in females, with the exception of breast cancer. In terms of the absolute number, the most common SPC was breast cancer (n = 1,202, OAR = 38.31, AER = −0.18) in female patients, while prostate cancer (n = 2,213, OAR = 61.33, AER = −7.87) was the most frequent SPC in males.

**Figure 2:**
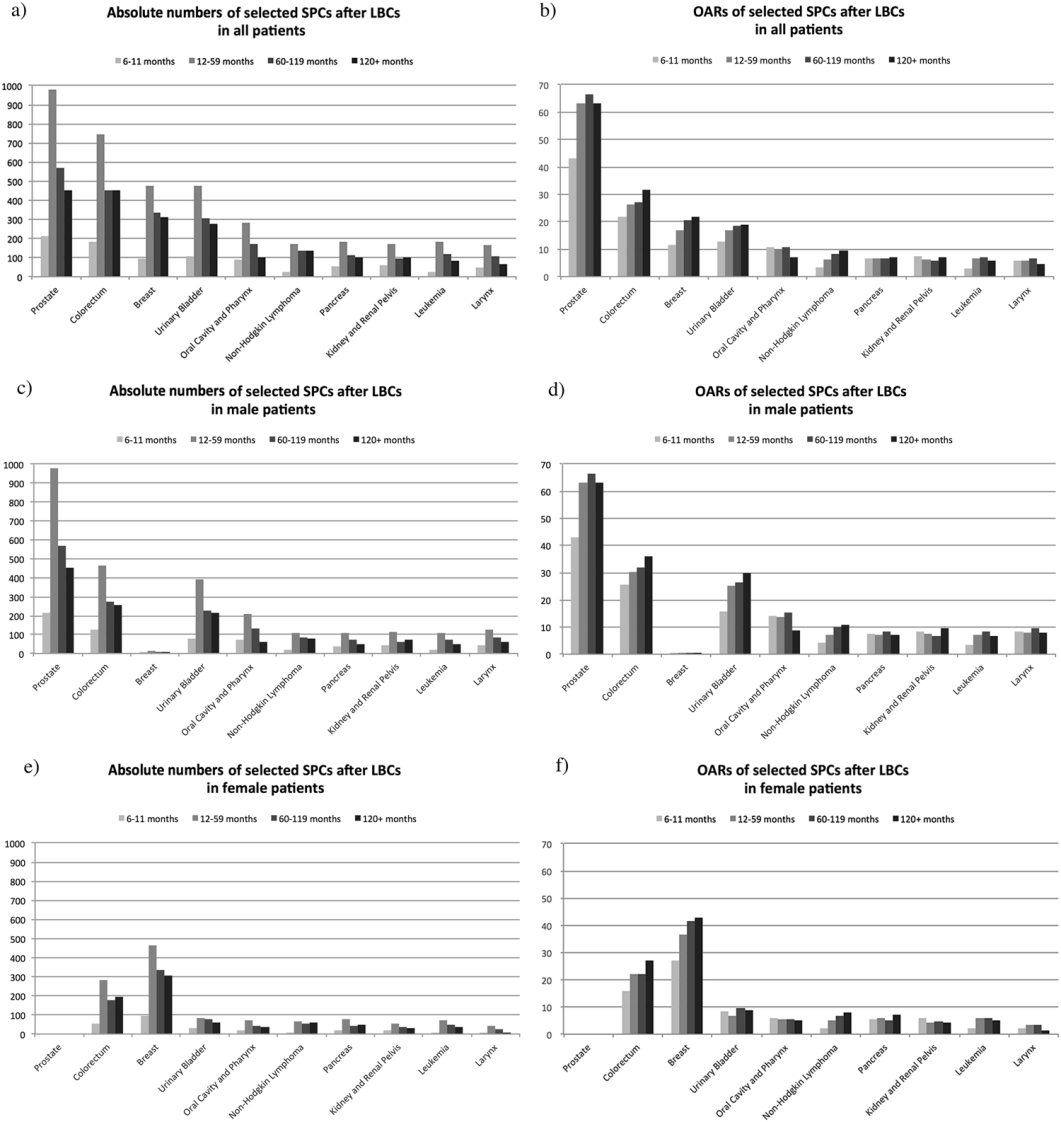
Absolute numbers and observed annual risks (OARs) of selected subsequent primary cancers (SPCs) after lung and bronchus caners (LBCs), by latency and gender.

Furthermore, incidences varied across different latency periods but with similarly fluctuant patterns. Presented in Fig. 2, Appendix Tables A1 and A2, 40.64% SPCs occurred in the early period (12–59 months) in terms of the absolute number, and this phenomenon persisted in all selected SPCs for both males or females. Moreover, OARs increased with follow-up and peaked at around 10 years after initial diagnosis (n = 2,786, OAR = 193.82, AER = 25.20), meaning that 1 out of every 51 lung and bronchus cancer patients would develop a SPC each year of this period. Furthermore, late latency (more than 120 months) was the most dangerous period in 4 selected SPCs among males and 4 among females.

### Lung and bronchus cancer following other cancers

Among all patients with non-lung bronchus cancer malignancies, overall OAR of subsequent lung and bronchus cancer was 27.90 (Table 3). The OAR was 37.86 per 10,000 PYR in males (n = 34,263, AER = 2.94), and 19.84 per 10,000 PYR in females (n = 22,216, AER = 2.55). According to the absolute numbers of subsequent lung cancer in each category, the top 10 cancer sites are shown in Fig. 1 and depicted in Table 3. The OAR ranged from low for melanoma (n = 1,665, OAR = 13.58, AER = −4.72) to high for larynx cancer (n = 3,037, OAR = 131.36, AER = 96.9). After gender stratification, the OAR for larynx cancer remained the highest in both males (n = 2,469, OAR = 131.18, AER = 93.31) and females (n = 568, OAR = 132.16, AER = 112.65).

**Table 3:** OARs, EARs, and AERs of subsequent LBCs after other cancers.

	Subsequent LBC after other cancers in all patients			Subsequent LBC after other cancers in male patients			Subsequent LBC after other cancers in female patients		
	Absolute numbers	OARs	AERs	Absolute numbers	OARs	AERs	Absolute numbers	OARs	AERs
		EARs			EARs			EARs	
All cancers excluding LBC	56,479	27.90	2.73	34,263	37.86	2.94	22,216	19.84	2.55
		25.17			34.92			17.29	
Prostate	12,522	34.96	−9.07	12,522	34.96	−9.07	—	—	—
		44.03			44.03				
Breast	8,843	18.53	0	94	37.19	0	8,749	18.43	0
		18.53			37.19			18.43	
Colorectum	7,022	29.85	−0.39	4,325	37.45	−2.15	2,697	22.52	1.31
		30.24			39.60			21.21	
Urinary Bladder	6,419	59.63	24.76	5,101	63.48	24.13	1,318	48.28	26.61
		34.87			39.35			21.67	
Oral Cavity and Pharynx	3,995	75.15	49.49	2,918	84.05	53.67	1,077	58.40	41.63
		25.66			30.38			16.77	
Larynx	3,037	131.36	96.90	2,469	131.18	93.31	568	132.16	112.65
		34.46			37.87			19.51	
Non-Hodgkin Lymphoma	2,077	28.37	6.52	1,167	30.67	5.16	910	25.89	8.00
		21.85			25.51			17.89	
Corpus Uteri	1,916	15.78	−5.01	—	—	—	1,916	15.78	−5.01
		20.79						20.79	
Melanoma	1,665	13.58	−4.72	995	16.52	−7.51	670	10.75	−2.01
		18.30			24.03			12.76	
Kidney and Renal Pelvis	1,320	28.81	3.54	893	32.25	2.03	427	23.55	5.83
		25.27			30.22			17.72	

Abbreviations: SPC, subsequent primary cancer; LBC, lung and bronchus cancer; OAR, observed annual risk; EAR, expected annual risk; AER, Annual excess risk.

**Figure 3:**
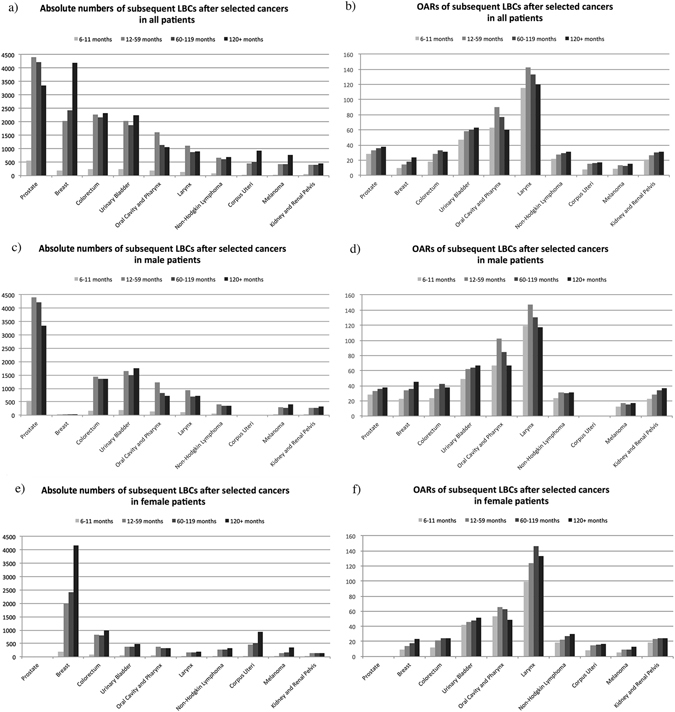
Absolute numbers and observed annual risks (OARs) of subsequent lung and bronchus caners (LBCs) after selected cancers, by latency and gender.

According to Fig. 3, Appendix Tables A3 and A4, most subsequent lung and bronchus cancers occur 10 years after initial diagnosis (n = 19,999, OAR = 28.21, AER = 2.26) in the field of absolute numbers. This was also observed in 8 sites in female patients and 4 sites in males. Regarding the OAR, however, the highest risk of subsequent lung cancer was found 60–119 months after initial diagnosis (n = 16,671, OAR = 28.88, AER = 4.04). This persisted in males (n = 10,522, OAR = 39.05, AER = 3.9) but shifted to a later period in female patients (after 120 months, n = 9,647, OAR = 22.31, AER = 2.01). The risk patterns during follow-up between the two genders were similar in that the highest OAR was observed in the late period (after 120 months) in 6 sites in males and 6 sites in females.

## Discussion

In the current population-based study, we reported the risks of SPCs after lung and bronchus cancer as well as the risks of lung and bronchus cancer after other malignancies. Recently, various SPCs have been reported after lung cancer in several centers^13–15^. In the present study, we examined absolute numbers, OARs, and occurrence patterns of the 10 most common SPCs, emphasizing the need for directed surveillance of these conditions.

Our results showed overall OAR of SPCs was 176.28 per 10,000 PYR, with AER of 23.97. In other words, 176 of 10,000 patients will develop a SPCs as a result of the history of lung cancer in one year, of which, 24 SPCs were additional occurrences beyond the number of primary cancers expected in the reference population. The highest OARs of SPC were observed in male prostate cancer patients and female breast cancer patients, consistent with the data of the general population, being the most frequently diagnosed malignancies of the male and female population^4^. Moreover, the risk of some SPCs varied by gender. This suggests the use of different surveillance strategies for male and female patients with primary lung and bronchus cancer as their initial diagnosis.

During the follow-up period, absolute numbers and OARs varied substantially across periods but with similar patterns. Consistent with previous studies^8, 9^, most SPCs occurred during the early period (12–59 months) in terms of the absolute number. In addition, we utilized the OAR to investigate the annual risks of SPCs in each period. The highest OAR was found in the late period (more than 120 months) either in overall SPCs or in selected malignancies, followed by the mid-term period (60–119 months). For instance, overall, 1 out of every 368 patients with lung cancer would develop a colorectal cancer each year during the follow-up period. Importantly, the risk rose to 1 in every 313 patients during 60–119 months and 1 in every 278 patients after 120 months. Showing an arbitrary mismatch with current follow-up strategies, which only emphasize early-phase screening, our results suggested that the frequency of surveillance of SPCs should be maintained throughout the entire follow-up period for each lung cancer survivor, and more intensely in the later period. Special attention needs to be paid to SPCs we described. Specific plans should be carefully framed by oncologists based on our findings and the NCCN guidelines of each malignancy^16–18^.

In this study, we also ranked our results by primary cancer sites with the 10 most frequent subsequent lung cancers and performed the analyses accordingly (Fig. 1 and Table 3). Primary cancers with the most subsequent lung cancer cases included prostate cancer among males and breast cancer among females, probably resulting from the already high incidences in the general population. The OARs of lung cancer following selected cancers yielded a 2- to 34-fold increase compared to the general incidence^4^. Furthermore, OARs were higher in males than in females among all selected non-genital malignancies. These data indicated that males with such primary cancers should be screened for lung cancer more frequently than females.

Although the highest absolute number and OAR of overall subsequent lung and bronchus cancer were observed for mid-term (60–119 months) and late (more than 120 months) periods, patterns varied by primary malignancy sites and gender. Such discrepancies might be attributed to different characteristics and therapies of primary cancers^14, 15^. In our present work, we summarized the risk patterns of subsequent lung and bronchus cancer by following 10 common cancers to provide evidence supporting the revision of the screening schedules. Consequently, surveillance of subsequent lung and bronchus cancer should be emphasized and given equal attention during each follow-up period, and a specific schedule should be made by oncologists according to the primary malignancies and NCCN guidelines of lung cancer^19^.

Previous studies have typically reported the risk ratios of the total observed number to the total expected number in different follow-up periods. However, the results were not comparable due to the different durations of each period (6 months vs. 48 months vs. 60 months vs. unknown), so the ratios of the total number were inappropriate to describe the risk. The current research has analyzed the annual incidence (OAR) of each subsequent primary cancer, resulting in more objective understanding of actual risk. Taking the SPCs that followed lung and bronchus cancer, for instance, although the AER of larynx cancer was increased by 3.96 per 10,000 PYR, their OAR was only 5.81. In other words, 1 out of 1721 lung cancer survivors will develop a larynx cancer each year, raised from 1/5405 in the reference population. However, the incidence of lung and bronchus cancer patients getting prostate cancer was 1/163 every year, which is still 10-fold that of larynx cancer, even though it is decreased from 1/145. In addition, we believed even the AER showed a decrease of 0.18 after lung cancer diagnosis, the breast cancer is still the top malignancy demanding surveillance on females.

The major highlight of this study is its population-based nature, utilizing a collection of a large number of patients with minimized selection biases and long-term follow-up. SEER*stat software also allowed us to analyze the risks of diseases in different follow-up latencies and thus helped us discover the patterns of SPCs. More importantly, the current study introduced absolute numbers and OARs to report the results of SPCs, which can provide a more accurate portrayal of the cancer burden in malignancy survivors.

Indeed, our study has a number of limitations mostly due to the sole application of the SEER database. First, distributions of histological types vary across different malignancies and with a good amount of unspecified records; therefore, we decided not to perform stratification by histological types in the analyses. Furthermore, the SEER database’s inaccessibility to known cancer risk factors, such as tobacco use, BMI, genetic data, chemotherapy, and the like, is also a major shortcoming that we have to address. These limitations prevented us from incorporating these factors into further analysis.

In conclusion, our findings refine the current surveillance strategy of SPCs for lung and bronchus cancer survivors during different follow-up periods as well as screening for subsequent lung and bronchus cancer in patients with other common malignancies.

## Electronic supplementary material

Supplementary Tables
