# Supplementary material for: The burdens of lung cancer involved multiple primary cancers and its occurring patterns–SEER Analysis between 1973 and 2006
Source: Sci Rep. 2017 Jul 25;7:6451. doi: 10.1038/s41598-017-06763-2 (PMC5527071; doi:10.1038/s41598-017-06763-2)
Supplement: Supplementary file 1 — Supplementary Tables [file 41598_2017_6763_MOESM1_ESM.doc]

**Title:** The burdens of lung cancer involved multiple primary cancers and its occurring patterns–SEER Analysis between 1973 and 2006.

**Running head**: Multiple cancers with lung cancer

**Anatomic site:** Lung

**Authors:** Rui Mao^1^, Tao Chen^1^, Fangyu Zhou^1^, Weili Jiang^2,3^, Xiaorong Yang^4^, Zisheng Ai^5^, Mu Li^1^, Linlin Qin^1^, Long Wang^1^, Ke Fei^1^*, Chang Chen^1^*

Rui Mao, Tao Chen, and Fangyu Zhou contributed equally to this work.

Ke Fei and Chang Chen contributed equally to this work.

**Authors’ Affiliation:**

1 Department of Thoracic Surgery, Shanghai Pulmonary Hospital, Tongji University School of Medicine, Shanghai, PR China.

2 Department of Epidemiology, School of Public Health, Fudan University, Shanghai, PR China.

3 Key Laboratory of Public Health Safety (Ministry of Education), Shanghai, PR China.

4 Department of Epidemiology and Biostatistics, Shandong University, Shandong, PR China.

5 Department of Medical Statistics, TongJi University School of Medicine, Shanghai, PR China.

**Key words:** lung cancer; subsequent primary cancers; risk factor; epidemiology; SEER.

**Correspondence to:**

Chang Chen, M.D.

Address: Department of Thoracic Surgery, Shanghai Pulmonary Hospital, Tongji University School of Medicine, 507 Zhengmin Road, Shanghai, China 200433

Tel.: +86 21 65115006 ext. 2074

Fax: +86 21 65111298

E-mail address: changchenc@hotmail.com

Ke Fei, M.D.

Address: Department of Thoracic Surgery, Shanghai Pulmonary Hospital, Tongji University School of Medicine, 507 Zhengmin Road, Shanghai, China 200433

Tel.: +86 21 65115006 ext. 2074

Fax: +86 21 65111298

E-mail address: [feikethoracic@sina.com](mailto:feikethoracic@sina.com)

**Acknowledgements:**

Founding: The work was supported by the projects from Science and Technology Commission of Shanghai Municipality (14DZ1941308).

Conflict of interest: None

**Author contribution:**

Rui Mao, Chang Chen, Ke Fei conceived the idea, Rui Mao, Tao Chen, Fangyu Zhou, Weili Jiang, Xiaorong Yang, Zisheng Ai analyzed the data, Rui Mao, Tao Chen, Fangyu Zhou wrote the main manuscript, Mu Li, Linlin Qin, Long Wang prepared the figures and tables，Rui Mao, Tao Chen, Fangyu Zhou, Weili Jiang, Xiaorong Yang, Zisheng Ai, Mu Li, Linlin Qin, Long Wang, Ke Fei, Chang Chen reviewed the article.

Appendix Table A.1. Absolute numbers of SPCs after LBCs by latency

Abbreviations: SPC, subsequent primary cancer; LBC, lung and bronchus cancer; m, months.

| Sites | SPCs after LBCs in all patients | | | |  | SPCs after LBCs in male patients | | | |  | SPCs after LBCs in female patients | | | |
| --- | --- | --- | --- | --- | --- | --- | --- | --- | --- | --- | --- | --- | --- | --- |
|  | 6-11 m | 12-59 m | 60-119 m | 120+ m |  | 6-11 m | 12-59 m | 60-119 m | 120+ m |  | 6-11 m | 12-59 m | 60-119 m | 120+ m |
| All cancers excluding LBC | 1,159 | 4,833 | 3,113 | 2,786 |  | 792 | 3,207 | 1,977 | 1,683 |  | 367 | 1,626 | 1,136 | 1,103 |
| Prostate | 214 | 979 | 568 | 452 |  | 214 | 979 | 568 | 452 |  | - | - | - | - |
| Colorectum | 183 | 744 | 451 | 454 |  | 128 | 464 | 273 | 257 |  | 55 | 280 | 178 | 197 |
| Breast | 96 | 476 | 338 | 313 |  | 1 | 11 | 5 | 4 |  | 95 | 465 | 333 | 309 |
| Urinary Bladder | 107 | 476 | 304 | 275 |  | 78 | 393 | 226 | 212 |  | 29 | 83 | 78 | 63 |
| Oral Cavity and Pharynx | 91 | 280 | 174 | 101 |  | 70 | 209 | 131 | 63 |  | 21 | 71 | 43 | 38 |
| Non-Hodgkin Lymphoma | 28 | 173 | 139 | 136 |  | 21 | 107 | 84 | 77 |  | 7 | 66 | 55 | 59 |
| Pancreas | 56 | 186 | 111 | 103 |  | 37 | 109 | 70 | 52 |  | 19 | 77 | 41 | 51 |
| Kidney and Renal Pelvis | 62 | 172 | 97 | 100 |  | 41 | 117 | 58 | 70 |  | 21 | 55 | 39 | 30 |
| Leukemia | 25 | 184 | 117 | 85 |  | 17 | 110 | 71 | 49 |  | 8 | 74 | 46 | 36 |
| Larynx | 48 | 168 | 108 | 68 |  | 41 | 124 | 82 | 58 |  | 7 | 44 | 26 | 10 |

Appendix Table A.2. OARs of SPCs after LBCs by latency

| Sites | SPCs after LBCs in all patients | | | |  | SPCs after LBCs in male patients | | | |  | SPCs after LBCs in female patients | | | |
| --- | --- | --- | --- | --- | --- | --- | --- | --- | --- | --- | --- | --- | --- | --- |
|  | 6-11 m | 12-59 m | 60-119 m | 120+ m |  | 6-11 m | 12-59 m | 60-119 m | 120+ m |  | 6-11 m | 12-59 m | 60-119 m | 120+ m |
| All cancers excluding LBC | 137.28 | 171.87 | 188.44 | 193.82 |  | 159.74 | 207.57 | 231.68 | 235.72 |  | 105.33 | 128.33 | 142.24 | 152.47 |
| Prostate | 43.16 | 63.37 | 66.56 | 63.31 |  | 43.16 | 63.37 | 66.56 | 63.31 |  | - | - | - | - |
| Colorectum | 21.68 | 26.46 | 27.30 | 31.58 |  | 25.82 | 30.03 | 31.99 | 36.00 |  | 15.78 | 22.10 | 22.29 | 27.23 |
| Breast | 11.37 | 16.93 | 20.46 | 21.78 |  | 0.20 | 0.71 | 0.59 | 0.56 |  | 27.26 | 36.70 | 41.70 | 42.71 |
| Urinary Bladder | 12.67 | 16.93 | 18.40 | 19.13 |  | 15.73 | 25.44 | 26.48 | 29.69 |  | 8.32 | 6.55 | 9.77 | 8.71 |
| Oral Cavity and Pharynx | 10.78 | 9.96 | 10.53 | 7.03 |  | 14.12 | 13.53 | 15.35 | 8.82 |  | 6.03 | 5.60 | 5.38 | 5.25 |
| Non-Hodgkin Lymphoma | 3.32 | 6.15 | 8.41 | 9.46 |  | 4.24 | 6.93 | 9.84 | 10.78 |  | 2.01 | 5.21 | 6.89 | 8.16 |
| Pancreas | 6.63 | 6.61 | 6.72 | 7.17 |  | 7.46 | 7.06 | 8.20 | 7.28 |  | 5.45 | 6.08 | 5.13 | 7.05 |
| Kidney and Renal Pelvis | 7.34 | 6.12 | 5.87 | 6.96 |  | 8.27 | 7.57 | 6.80 | 9.80 |  | 6.03 | 4.34 | 4.88 | 4.15 |
| Leukemia | 2.96 | 6.54 | 7.08 | 5.91 |  | 3.43 | 7.12 | 8.32 | 6.86 |  | 2.30 | 5.84 | 5.76 | 4.98 |
| Larynx | 5.69 | 5.97 | 6.54 | 4.73 |  | 8.27 | 8.03 | 9.61 | 8.12 |  | 2.01 | 3.47 | 3.26 | 1.38 |

Abbreviations: OAR, observed annual risk; SPC, subsequent primary cancer; LBC, lung and bronchus cancer; m, months.

Appendix Table A.3. Absolute numbers of subsequent LBCs after other cancers by latency

Abbreviations: SPC, subsequent primary cancer; LBC, lung and bronchus cancer; m, months.

| Sites | Subsequent LBCs after other malignancies in all patients | | | |  | Subsequent LBCs after other malignancies in male patients | | | |  | Subsequent LBCs after other malignancies in female patients | | | |
| --- | --- | --- | --- | --- | --- | --- | --- | --- | --- | --- | --- | --- | --- | --- |
|  | 6-11 m | 12-59 m | 60-119 m | 120+ m |  | 6-11 m | 12-59 m | 60-119 m | 120+ m |  | 6-11 m | 12-59 m | 60-119 m | 120+ m |
| All cancers excluding LBC | 2,148 | 17,661 | 16,671 | 19,999 |  | 1,504 | 11,885 | 10,522 | 10,352 |  | 644 | 5,776 | 6,149 | 9,647 |
| Prostate | 549 | 4,394 | 4,233 | 3,346 |  | 549 | 4,394 | 4,233 | 3,346 |  | - | - | - | - |
| Breast | 192 | 2,023 | 2,437 | 4,191 |  | 3 | 30 | 27 | 34 |  | 189 | 1,993 | 2,410 | 4,157 |
| Colorectum | 242 | 2,268 | 2,175 | 2,337 |  | 162 | 1,438 | 1,369 | 1,356 |  | 80 | 830 | 806 | 981 |
| Urinary Bladder | 254 | 2,044 | 1,868 | 2,253 |  | 199 | 1,653 | 1,492 | 1,757 |  | 55 | 391 | 376 | 496 |
| Oral Cavity and Pharynx | 196 | 1,605 | 1,144 | 1,050 |  | 143 | 1,220 | 825 | 730 |  | 53 | 385 | 319 | 320 |
| Larynx | 148 | 1,114 | 870 | 905 |  | 125 | 937 | 692 | 715 |  | 23 | 177 | 178 | 190 |
| Non-Hodgkin Lymphoma | 88 | 678 | 623 | 688 |  | 52 | 410 | 340 | 365 |  | 36 | 268 | 283 | 323 |
| Corpus Uteri | 35 | 459 | 500 | 922 |  | - | - | - | - |  | 35 | 459 | 500 | 922 |
| Melanoma | 41 | 435 | 417 | 772 |  | 30 | 290 | 261 | 414 |  | 11 | 145 | 156 | 358 |
| Kidney and Renal Pelvis | 49 | 396 | 407 | 468 |  | 33 | 262 | 277 | 321 |  | 16 | 134 | 130 | 147 |

Appendix Table A.4. OARs of subsequent LBCs after other cancers by latency

| Sites | Subsequent LBCs after other malignancies in all patients | | | |  | Subsequent LBCs after other malignancies in male patients | | | |  | Subsequent LBCs after other malignancies in female patients | | | |
| --- | --- | --- | --- | --- | --- | --- | --- | --- | --- | --- | --- | --- | --- | --- |
|  | 6-11 m | 12-59 m | 60-119 m | 120+ m |  | 6-11 m | 12-59 m | 60-119 m | 120+ m |  | 6-11 m | 12-59 m | 60-119 m | 120+ m |
| All cancers excluding LBC | 21.60 | 27.65 | 28.88 | 28.21 |  | 30.62 | 38.38 | 39.05 | 37.41 |  | 12.79 | 17.55 | 19.98 | 22.31 |
| Prostate | 28.71 | 33.11 | 35.81 | 37.94 |  | 28.71 | 33.11 | 35.81 | 37.94 |  | - | - | - | - |
| Breast | 9.48 | 14.18 | 17.90 | 23.52 |  | 22.69 | 33.71 | 36.19 | 44.79 |  | 9.39 | 14.06 | 17.80 | 23.43 |
| Colorectum | 17.92 | 28.35 | 33.15 | 30.70 |  | 23.59 | 35.59 | 42.29 | 37.83 |  | 12.06 | 20.96 | 24.25 | 24.35 |
| Urinary Bladder | 47.08 | 57.96 | 60.00 | 62.84 |  | 48.83 | 61.94 | 64.00 | 66.88 |  | 41.69 | 45.57 | 48.09 | 51.78 |
| Oral Cavity and Pharynx | 62.86 | 90.39 | 77.24 | 60.10 |  | 67.12 | 102.39 | 84.76 | 66.74 |  | 53.66 | 65.90 | 62.82 | 48.98 |
| Larynx | 115.71 | 142.67 | 133.34 | 120.54 |  | 119.40 | 146.93 | 130.28 | 117.49 |  | 99.09 | 123.70 | 146.79 | 133.60 |
| Non-Hodgkin Lymphoma | 21.51 | 26.92 | 28.98 | 30.67 |  | 23.93 | 31.13 | 30.62 | 31.45 |  | 18.77 | 22.31 | 27.23 | 29.84 |
| Corpus Uteri | 8.00 | 14.86 | 15.68 | 17.00 |  | - | - | - | - |  | 8.00 | 14.86 | 15.68 | 17.00 |
| Melanoma | 9.01 | 13.22 | 12.41 | 14.99 |  | 12.46 | 17.04 | 15.48 | 17.28 |  | 5.13 | 9.13 | 9.31 | 12.99 |
| Kidney and Renal Pelvis | 21.01 | 26.38 | 30.04 | 31.36 |  | 22.70 | 28.24 | 33.70 | 36.74 |  | 18.22 | 23.36 | 24.40 | 23.76 |

Abbreviations: OAR, observed annual risk; SPC, subsequent primary cancer; LBC, lung and bronchus cancer; m, months.
